# Bilateral synchronous testicular cancer: A case report

**DOI:** 10.1016/j.ijscr.2022.107870

**Published:** 2023-01-04

**Authors:** Jeremy Thompson Ginting, Bungaran Sihombing, Syah Mirsya Warli, Ginanda Putra Siregar, Fauriski Febrian Prapiska

**Affiliations:** a Department of Urology, Faculty of Medicine, Universitas Indonesia - Haji Adam Malik General Hospital, Indonesia; b Division of Urology, Department of Surgery, Faculty of Medicine, Universitas Sumatera Utara - Haji Adam Malik General Hospital, Indonesia; c Department of Urology, Universitas Sumatera Utara Hospital, Universitas Sumatera Utara, Indonesia

**Keywords:** TC, testicular cancer, GCT, germ cell tumor, NSGCT, non-seminoma germ cell tumor, BTGCT, bilateral germ cell tumor, Testicular cancer, Germ cell tumor, Synchronous bilateral testicular cancer

## Abstract

**Introduction and importance:**

Testicular cancer is the most common type of malignancy in young adult males, accounting for 1 % of all cancer diagnosis in men and 5 % of all urologic tumors. It is one of the malignancies with the highest cure rate. Bilateral germ cell tumor of the testicles is rare, representing only 1 % of all new cases of testicular cancer, around 30 % of which occur synchronously. Interestingly, there is not yet an occurrence where the bilateral synchronous testicular cancer has different histopathological type.

**Case presentation:**

In this paper, we performed bilateral radical orchiectomy in different occasion, followed by adjuvant chemotherapy (BEP regiment).

**Clinical discussion:**

Since the clinical result is excellent, this finding could be a breakthrough in testicular cancer study.

A cellular communication between different cancer cell type through chemokine which could affect response to chemotherapy.

**Conclusion:**

Treatment with surgery and chemotherapy is well tolerated and received. A further specific clinical study needs to be performed to investigate this finding in the future.

## Highlights

• Bilateral synchronous testicular cancer is a rare case.
• There is not yet an occurrence where the bilateral synchronous testicular cancer has different histopathological type.
• There is no consensus on treatment for bilateral synchronous testicular cancer.
• Standard treatment for all testicular tumor in adult is radical inguinal orchiectomy.
• Treatment with surgery and chemotherapy is well tolerated and received and give good result.

## 1 Introduction

Testicular cancer is the most common type of malignancy among males in the 15–35-year-old range [1]. It accounts for 1 % of all cancer diagnosis in men [1], [2]. Around 95 % of testicular malignancies are germ cell tumors [3]. Incidence of bilateral testicular cancer is around 1–5 % of all testicular cancer [4]. About one-third of bilateral testicular cancer is diagnosed as synchronous, while the other two-thirds are metachronous [4]. Testicular malignancies has excellent prognosis with treatment. Cure is achievable in 95 % of all patients and 80 % to those with metastatic disease with adequate treatment [5]. This number however significantly decreases in bilateral testicular cancer, especially when initial diagnosis was made in advanced stages.

Here we report a case of a 31-year-old male with synchronous bilateral testicular cancer with metastasizes to abdominal and cervical nodes. This patient has a different tumor histopathology between his right and left testis. We reviewed the diagnosis, and treatment, and compare it to other published case reports of bilateral testicular malignancies. Patient gives his consent for this study.

This case report was made following SCARE 2020 Guideline and submitted after completed SCARE 2020 checklist [6].

## 2 Presentation of case

A thirty-one-year-old Indonesian male, with no significant medical history, presented to our facility with complaints of a lump in the right testicular area that he noticed about 2 months earlier. The patient was in his usual state of health until 4 months earlier when he began to notice left testicular swelling. There was no record of drug medication and no family history of malignancy. Patient was not a smoker.

A workup was done and the patient went through a radical orchidectomy of his left testis in another facility 2 months before coming to our facility. Pathological examination of the orchiectomy specimen of the left testis revealed a seminoma and teratoma of the testis, which led to a diagnosis of non-seminoma germ cell testicular tumor. It was noted in the pathological examination that the tumor has lymphovascular invasion. A few weeks after the procedure was completed, the patient noticed a new lump in right testicular area. The patient reported that the mass was initially small in size and exponentially grew into a larger, denser lump. Pain was absent. During this time period he also noted a 10 kg of unintentional weight loss.

The patient has no significant systemic comorbidities. History of cigarette consumption was absent. The patient has a history of left testicular torsion 8 years prior. A left orchidopexy was performed right after the diagnosis. The surgery was performed by a senior urologist at our hospital.

**Fig. 1:**
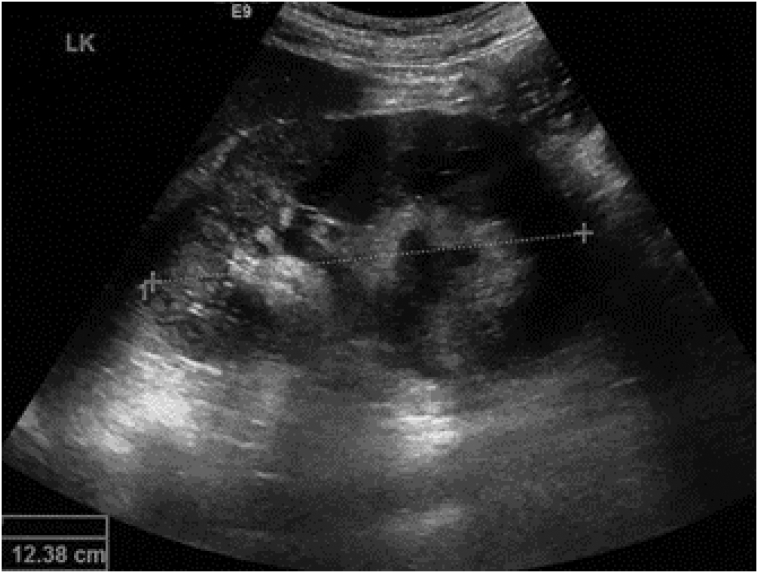
Ultrasound examination of the left kidney illustrating moderate hydronephrosis.

**Fig. 2:**
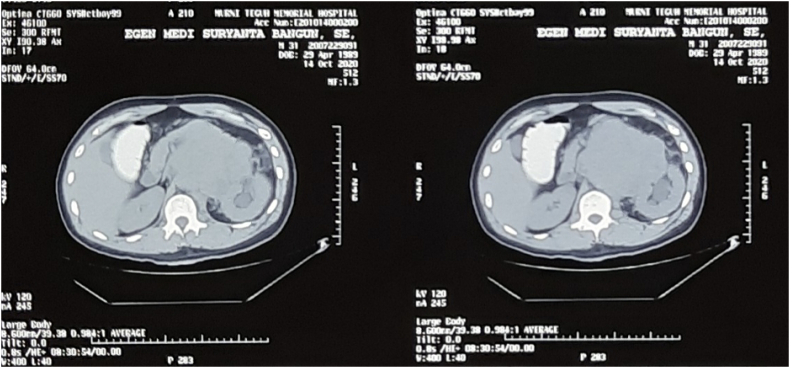
CT scan with contrast prior to patient first orchiectomy (right testis) and before BEP administration.

Physical examination showed an enlarged cervical lymph node on his left neck with a diameter of around 4 cm. We performed an abdomen CT scan prior to both of his surgical procedures, which revealed a 13 × 9 × 14 cm solid mass on the level of the left kidney, suggestive of a retroperitoneal lymph node. The mass was revealed to have shifted the aorta to the right and the pancreas anteriorly. Mild hydronephrosis was also observed due to compression of left kidney hilus (Fig. 1). Otherwise, the CT scan is unremarkable. After orchiectomy on his left testis and before his orchiectomy on his right testis, we performed a non-contrast CT scan (Fig. 2), which revealed an enlargement of retroperitoneal lymph node 7 cm in size and the CT scan with contrast reveals the size of 9 cm in diameter. This difference may be due to the higher sensitivity with the use of contrast. Chest radiograph showed no abnormal findings. Laboratory examinations prior to his second surgery elevated b-hGC (100.89 mIU/mL), AFP (72.04 ng/mL), and LDH (861 IU/L). Before the first operation, tumor markers were not checked.

**Fig. 3:**
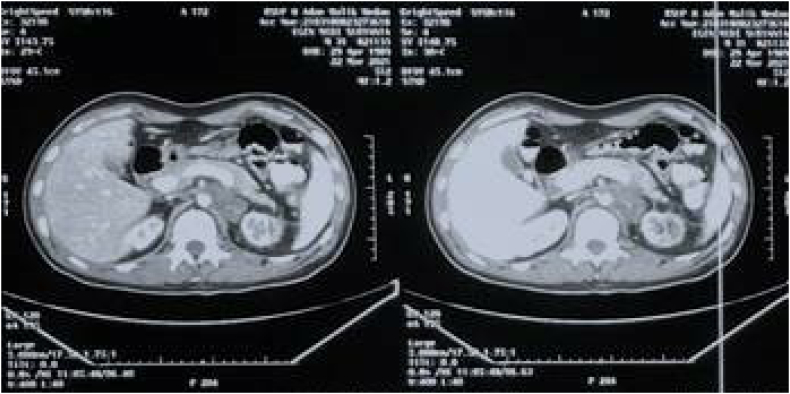
CT scan with contrast 3 months after BEP administration.

**Fig. 4:**
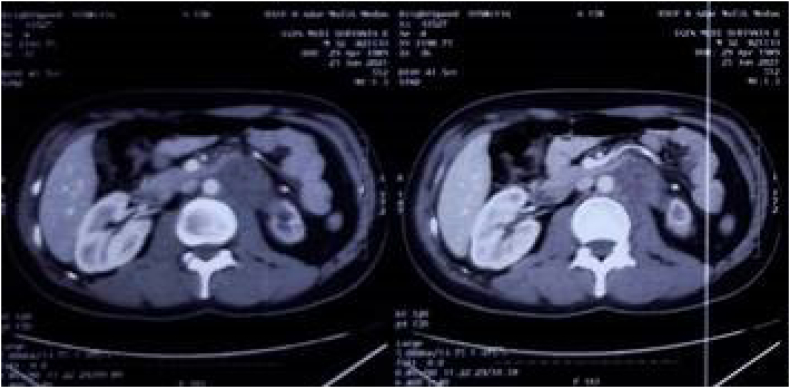
CT scan with contrast 6 months after BEP administration.

**Fig. 5:**
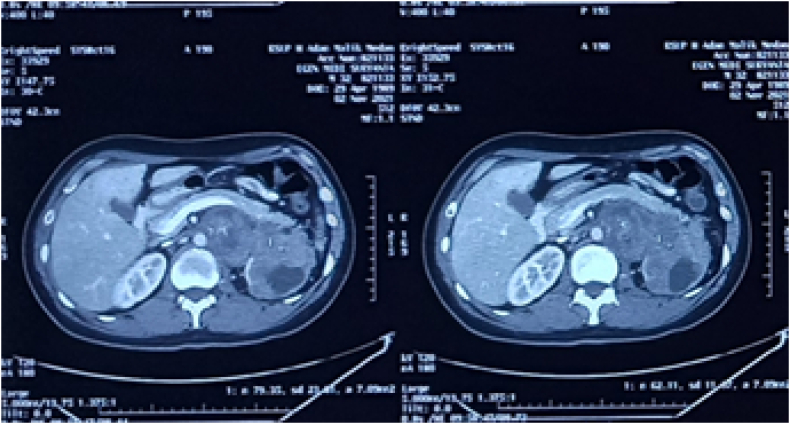
CT scan with contrast.

**Fig. 6:**
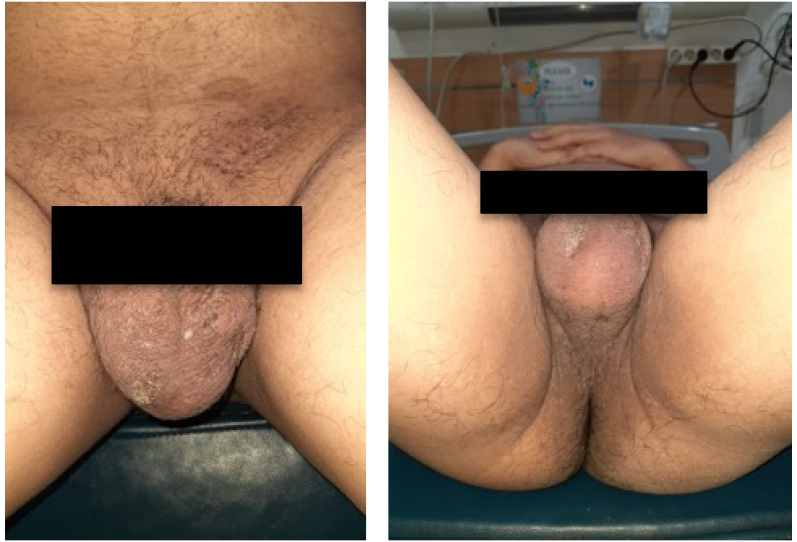
Right testicle enlargement.

**Fig. 7:**
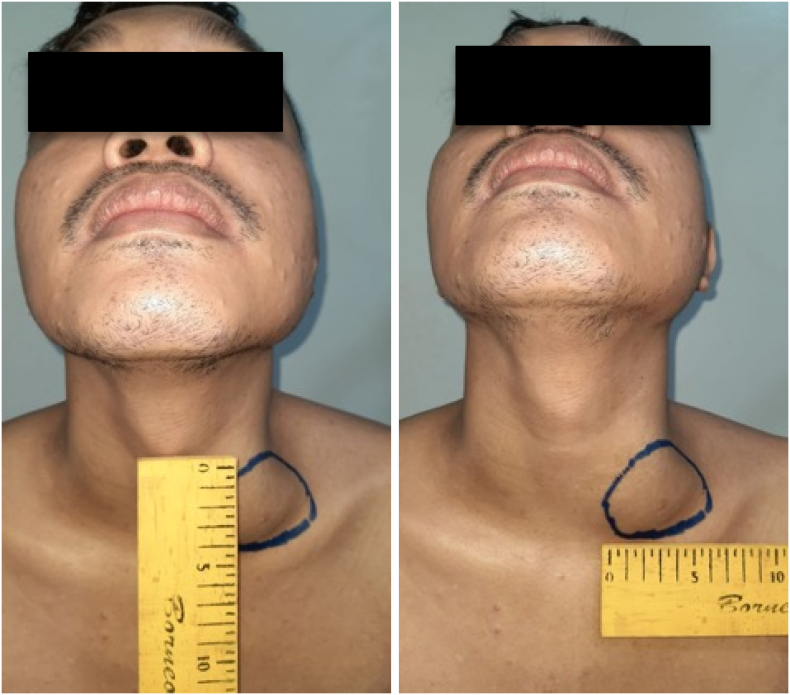
Enlarged lymph node.

**Fig. 8:**
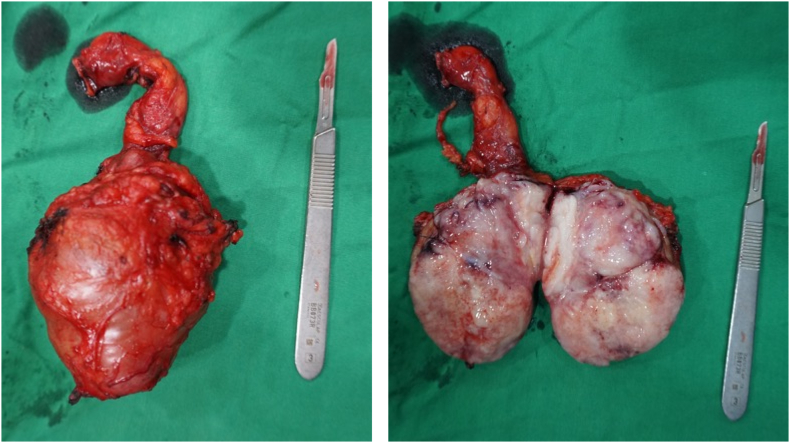
Post radical orchiectomy of the right testicle.

**Fig. 9:**
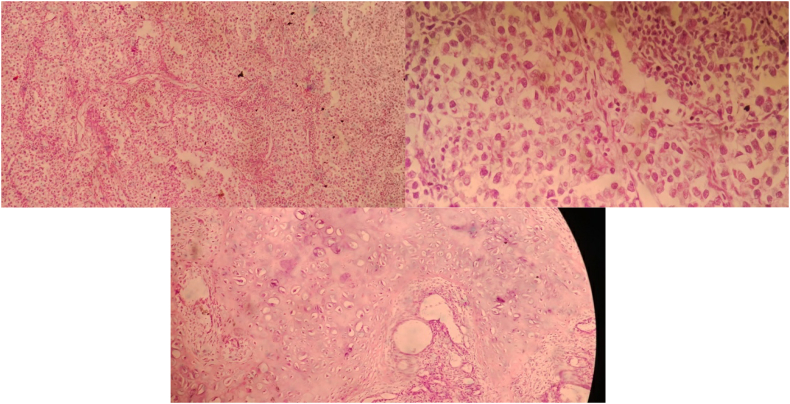
Histopathology examination result.

For his current complaint, the patient underwent a right orchiectomy done in our facility. Pathological examination of this orchiectomy specimen revealed a seminoma of the testis, histopathological result can be seen in Fig. 9. On this basis, the patient was diagnosed with a bilateral synchronous testicular cancer. Bilateral testicular tumors that occur simultaneously are termed synchronous tumors, while those occurring at different times are termed metachronous tumors. Due to findings indicating that the cancer has metastasized to a distant lymph node and due to it being a bilateral tumor, systemic treatment was done. What needs to be considered is that the target of therapy and the choice of therapy must be in accordance with the patient in order to achieve the best adherence, tolerability and effectiveness. Once the patient had a definitive diagnosis, he began 4 cycles of chemotherapy with cisplatin, bleomycin, and etoposide (BEP regimen). Upon evaluation, 3 months post chemotherapy with BEP regimen, the mass on his left cervical lymph node was no longer palpable, and his retroperitoneum nodule shrunk to 2 cm in diameter (Fig. 3) and Laboratory examinations showed normal tumor markers of testicular cancer. After 6 months, the patient was re-evaluated, the results of the CT scan with contrast showed tumor nodule regrowth to the kidneys and liver (Fig. 3). We decided to start chemotherapy with a regimen of Paclitaxel, Ifosfamide and Cisplatin (TIP). 3 months after being given TIP, the patient was re-evaluated and the CT scan results showed a significant reduction in the number of tumor nodules (Fig. 5) as well as Laboratory examinations showed normal tumor markers of testicular cancer. One and a half year after the operation and chemotherapy, no recurrence was observed. The patient is in good clinical condition, Karnofsky is 90 and his food and fluid intake is good. After several cycles of chemotherapy, the patient also noticed an improvement. Unfortunately, the patient refused RPLND. Currently the patient is undergoing second line chemotherapy with TIP due to an increase in the size of the retroperitoneal nodules (Fig. 4, Fig. 6, Fig. 7, Fig. 8).

## 3 Clinical discussion

Testicular cancer is the most common type of malignancy in young adult males, comprising of 1 % of all male cancer diagnosis [2]. Incidence of bilateral testicular cancer constitutes 1–5 % of all testicular cancer [4]. Bilateral testicular tumors are either metachronous or synchronous. Synchronous tumors occur simultaneously, in which both tumors become apparent at the time of diagnosis or within the first two months after establishment of initial diagnosis [4]. Incidence of synchronous tumors is around one-third of all bilateral testicular cancer [4], [7]. Unlike most cancers that occur in adulthood, the incidence of testicular cancer does not increase with age. Testicular cancer (TC) has a distinctive age distribution, in which it is commonly diagnosed among younger men, aged 15–40 years [2]. Peak age of occurance is 35–39 years for seminoma and 25–29 years for non-seminoma [8]. Our patient is a 31-year-old man, within the common age group for testicular cancer. The diagnosis for his second tumor (left testicle) was established within two months of his first diagnosis (right testicle). On this basis, our patient is diagnosed with a synchronous bilateral testicular cancer.

Known risk factors for testicular cancers include cryptorchidism, prior testicular germ cell tumor (TGCT), having a father with TGCT, having a brother with TGCT, and higher body height. Prior testicular germ cell tumor (TGCT) and higher body height are the risk factors that are found in this patient's case. Among these factors, the greatest relative risk is having a brother with testicular TGCT, increasing the individual's risk by approximately 10 times (RR 7.55–12.74, 95 % CI), followed by history of cryptorchidism (RR 4.3, 95 % CI) [7]. These known factors were under the prevailing hypothesis that the etiology of testicular cancer is largely determined *in utero*. Several studies have identified genetic markers likely linked with testicular cancer, with the strongest associated observed for a single-nucleotide polymorphism in the 12q21 locus. However, it was found that among first degree relatives, polymorphism of locus on comprises 11 % of the risk of developing testicular cancer. Therefor it was hypothesized, that perinatal risk factors also have a role in the development of testicular cancer. Two meta-analyses found that inguinal hernia, low birth order, small sibship size, maternal bleeding and being a twin to have an association with increased risk of testicular cancer [8]. Risk factors for bilateral testis cancer, are the same as for unilateral testis cancer [7]. Our patient has no notable history related to these risk factors. Occupational factors were also not notable.

Almost all testicular cancer is a germ cell tumor (GCT) [9]. Diagnosis is commonly differentiated into seminomatous and non-seminomatous germ cell tumors, accounting for approximately 40 % and 60 % of all testicular cancer, respectively [1]. Non-seminoma GCTs (NSGCTs) are divided into found subtypes: teratoma, embryonal, yolk sac, and teratoma [9]. Mixed germ cell tumors, containing more than one germ cell component are much more common than the pure histologic form. In synchronous BTGTCs, bilateral seminoma, bilateral non-seminoma, non-germ cell tumor, and tumor with different histology comprise of 48 %, 15 %, 22 % and 15 % of diagnosis, respectively [5], [10]. Most patients with TC have a chief complaint of a painless testicular mass [11]. If the disease has metastasized at the time of diagnosis, the patient might have other complaint on a distant location to the testes. On physical examination we found an enlarge lymph node on the right cervical area, although our patient did not specifically complain of a mass on the neck. The findings in our patient's CT scan also suggest a more extensive retroperitoneal involvement, compressing surrounding organs. Upon suspicion of a testicular mass, the most accessible test to be ordered is a scrotal ultrasound. Findings of an intratesticular lesion, usually illustrated as a hypoechoic, heterogenous intratesticular mass with irregular margins is suggestive of a TC [11], [12]. For detection of a testicular malignancy, an ultrasound examination has a sensitivity and specificity of 92–98 % and 95–99 % respectively [12]. In our patient, USG illustrated a heterogenous intratesticular mass with irregular margins with various echogenicity, which is suggestive of TC. Several laboratory workups can be ordered to support the diagnosis of TC, such as lactate dehydrogenase (LDH), beta-human chorionic gonadotropin (hCG), and alpha-fetoprotein (AFP) [11]. Laboratory examinations prior to orchiectomy on our patient illustrated elevated levels of LDH, AFP, and hCGs. Around 25 % of patients with seminoma have elevated hCG levels, as is in our patient who had a hCG level of 100 mIU/ml [1], [9]. However, hCG levels may also be elevated in NSGCTs. AFP levels are almost never elevated in pure seminoma, but is elevated in 60 % of NSGCTs [1], [9]. Teratomatous glands also cause elevation of AFP [13]. Additional to this workup, pathological examinations of orchiectomy tissue should be ordered. Pathological tissue examination for our patient revealed a non-seminoma (mixed type comprising of seminoma) and teratoma on the left testicle and seminoma on the right testicle. On the basis of both examinations, our patient is consistent with a synchronous non seminoma BTGCT.

Staging in NSGCTs uses comprehensive tumor/node/metastasis staging system by the American Joint Committee on Cancer and the International Union Against Cancer for each tumor [11]. Under this staging system, for his left tesis, our patient would be classified as follows:

• pT2 – Tumor extending through the tunica albuginea with involvement of the tunica vaginalis
• N3 – Metastasis with a lymph node mass more than 5 cm in greatest dimension
• M1a – Non-regional nodal or pulmonary metastasis
• S2 – LDH 1.5–10× upper limit of normal

With this classification, our patient classified as stage IIIB NSGCT (mixed germ cell of seminoma and teratoma) for his left testis. The patient was also diagnosed with the same stage of seminoma NSGCT on his right testis. Under the staging system of the International Germ Cell cancer Collaborative Group classification, our patient is classified as intermediate risk, giving him a 5-year survival rate of around 72 % [14]. This number is considerably lower for patients with synchronous bilateral tumor, especially those with advance diseases on first diagnosis [15]. Studies on prognosis are quite scarce since reported cases are little to begin with. A study by Hentrich, et al., on 1180 with testicular tumors reported an average disease-free survival of 37 months in 14 patients with bilateral synchronous tumor [16].

Standard treatment for all testicular tumor in adult is radical inguinal orchiectomy. In selected patients with bilateral tumors, conservative procedures in a form of partial orchiectomy can be considered, only when the tumor volume is less than 30 % of the testicular volume or smaller than 2 cm and is away from testicular vasculature [16]. Up to date there are no consensus on treatment for bilateral synchronous testicular cancer, thus treatments are done on a case-by-case basis. In a case report done by Anastasiou, et al [10], summarizing previous case reports for synchronous GCTC with different histopathology, most reported cases area treated with bilateral orchiectomy and several 2–4 rounds of BEP regimen chemotherapy. Our patient underwent a bilateral orchiectomy followed by a regimen of BEP × 4 cycles [11]. All patients with NSGCT with persistent retroperitoneal mass greater than 1 cm should undergo a postchemotherapy retroperitoneal lymph node dissection (RPLND) and treatment should only be done when STMs (Serum Tumor Marker) are negative [11]. Our patient did not exert findings of a retroperitoneal mass in the first place, hence we did not do a postchemotherapy RPLND.

Our patient needs follow up to assess relapses and treatment-related sequele. Most TC recurrences occur within 2 years. Treatment-related sequelae include hearing loss form cisplatin-based chemotherapy, increased risk of death from cardiovascular and pulmonary disease after chemotherapy, as well as increased incidence of non-germ cell malignancies in TC survivors treated with a cisplatin-based chemotherapy compared to the general population. Published case reports or case series reporting survival rates and follow up reports of patients with synchronous GCTC with different histopathology are scarce. Two case reports of earlier stages of synchronous GCTC with different histopathology showed no recurrences after 1–2 years of follow up [10], [17]. Follow up should include physical examination, serum marker measurement, chest radiography, scrotal USG and CT abdomen [15]. Follow up after 1,5 years showed no recurrences in our patient. Patients with bilateral orchiectomy are also suggested to be given hormone replacement therapy to treat potential androgen insufficiency [4]. This case report is contrast from a case report presented by Manolitsis I. et al. that shows a 44-year-old man with a right radical inguinal orchiectomy was performed with a pathology report showing seminoma staged T1a according to the current TNM (tumor, node, metastasis) classification, mature teratomas, and lesions of germ cell neoplasia *in situ*. A month later, the first patient, after completing 2 cycles of chemotherapy with objective clinical, biochemical, and imaging improvement in all metastatic lesions (thorax, retroperitoneum), suffered from an episode of massive hematochezia and died [18]. Pure choriocarcinoma has an aggressive oncological behavior with high metastatic capacity such as choriocarcinoma syndrome which causes bleeding in metastatic sites and significant morbidity and mortality 3; Alvarado/Hernandez et al. reported a series of 15 patients with choriocarcinoma as a pure or predominant component, finding 100 % of cases with metastases (pulmonary 66 %, hepatic 60 %, brain 20 % gastrointestinal tract 13 % and renal 6 %) 4; There are few studies or reports in the literature of retroperitoneal bleeding or better known as Wunderlich syndrome in association with testicular cancer, finding publications such as Yee-Huang Ku et al., in 2018 reporting a case of spontaneous retroperitoneal bleeding secondary to testicular choriocarcinoma, 3 or as published by Huang et al. reporting another case of choriocarcinoma-type testicular carcinoma with metastatic retroperitoneal involvement associated with Wunderlich syndrome 5; In Salgado et al. case, the high aggressiveness of this type of testicular tumors is clearly evidenced, documenting metastasis to the kidney and mesentery, confirming with the histopathological result the commitment to choriocarcinoma, patient was treated in intensive care unit for hemorrhagic shock grade IV, acute liver failure and acute renal failure, presenting poor evolution with subsequent death at 24 h [19]. Venkata et al. also presented a 23 year old male with increasing abdominal pain, diarrhea, episodic vomiting for 3 weeks, the CT scan of the abdomen revealed a caecal mass with multiple metastases to liver, lungs and abdominal lymph nodes and biopsies from the mass were reported as poorly differentiated metastatic carcinoma. The patient responded well to chemotherapy with a drop in HCG levels; however, hospital course was complicated with tumor lysis syndrome and neutropenic sepsis, which progressed to multi-organ dysfunction. He required mechanical ventilatory support along with vasopressors for blood pressure support. As a result of deterioration, the patient expired 36 h later. Even though his cancer burden was responding to chemotherapy, he unfortunately succumbed from complications of the treatment [20]. The majority bilateral seminoma, which present with a low stage at diagnosis, and mixed histology tumors have a good overall survival. On the other hand, cases with bilateral non-seminoma histology are associated with poor prognosis and high stage at presentation. Appropriate adequate therapy is very influential on patient outcomes.

## 4 Conclusion

This article summarizes a case of synchronous bilateral non-seminoma testicular cancer on a young male. This disease is quite rare, especially given the histology type. Treatment with surgery and chemotherapy is well tolerated and received. Since most TC patients are young men, further studies and efforts maximizing treatment efficacy while minimizing the morbidity associated with treatment is needed.

This article summarizes a case of synchronous bilateral non-seminoma testicular cancer on a young male. Treatment with surgery and chemotherapy is well tolerated and received and give good result.

## Informed consent

Written informed consent was obtained from the patient for publication of this case report and accompanying images. A copy of the written consent is available for review by the Editor-in-Chief of this journal on request.

## Ethical approval

The study protocol is compliant with ethical standards and approved by local ethical committee (261/KEPK/USU/2022).

## Source of funding

This study is a self-funding study and received no financial support from any external sources, public or private sectors.

## Author contribution

Jeremy Thompson Ginting:

- Conception and design
- Analysis and interpretation of the data
- Drafting of the article
- Statistical expertise
- Provision of study materials or patients

Bungaran Sihombing:

- Conception and design
- Analysis and interpretation of the data
- Drafting of the article
- Critical revision of the article for important intellectual content
- Final approval of the article

Syah Mirsya Warli:

- Conception and design
- Drafting of the article
- Critical revision of the article for important intellectual content
- Final approval of the article

Ginanda Putra Siregar

- Drafting of the article
- Critical revision of the article for important intellectual content
- Final approval of the article

Fausriski Febrian Prapiska

- Drafting of the article
- Critical revision of the article for important intellectual content
- Statistical expertise
- Final approval of the article

## Guarantor

Bungaran Sihombing, Syah Mirsya Warli, Ginanda Putra Siregar, Fausriski Febrian Prapiska.

## Research registration number

1. Name of the registry: Komite etik penelitian Kesehatan, FK USU, Medan
2. Unique Identifying number or registration ID: No. 261/KEPK/USU/2022

## Declaration of competing interest

There is no conflict of interest in this paper work.
